# Prevalence and patterns of tobacco smoking among Chinese adult men and women: findings of the 2010 national smoking survey

**DOI:** 10.1136/jech-2016-207805

**Published:** 2016-09-22

**Authors:** Shiwei Liu, Mei Zhang, Ling Yang, Yichong Li, Limin Wang, Zhengjing Huang, Linhong Wang, Zhengming Chen, Maigeng Zhou

**Affiliations:** 1 National Center for Chronic and Noncommunicable Disease Control and Prevention, Chinese Center for Disease Control and Prevention, Beijing, China; 2 Clinical Trial Service Unit & Epidemiological Studies Unit (CTSU), Nuffield Department of Population Health, University of Oxford, Oxford, UK

**Keywords:** SMOKING, Tobacco, EPIDEMICS, SURVEILLANCE

## Abstract

**Background:**

China consumes about 40% of the world's cigarettes, predominantly by men, following a large increase in recent decades. We assess sex-specific prevalence and changing patterns of smoking in Chinese adults in the current decade.

**Methods:**

A nationally representative survey of smoking was conducted in 2010 among 100 000 Chinese adults aged ≥18 years, using a multistage stratified cluster sampling method. Information on smoking frequency, type, amount, age started and quitting was collected. Sex-specific standardised prevalence and means were analysed and compared with estimates in the 1996 national survey.

**Results:**

In Chinese men aged ≥18, 62.4% were ever-smokers in 2010, including 54.0% current smokers and 8.4% ex-smokers. The smoking prevalence was higher in rural than in urban men (63.9% vs 58.4%). In younger men, the age to start smoking was earlier and exclusive cigarette use was much higher. Among current smokers, only 17.3% intended to quit. Compared with a similar survey in 1996 among adults aged 30–69, more smokers had quit in 2010 than in 1996 (11.0% vs 4.2%), but the number of cigarettes smoked per current smoker was higher (17.9 vs 15.2). In Chinese women, only 3.4% ever smoked and there has been a large intergenerational decrease in smoking uptake rates. In 2010, there were 318 million current smokers in China, consuming an estimated 1740 billion cigarettes.

**Conclusions:**

The prevalence of smoking remained extremely high in men, but low and falling in Chinese women. Tobacco smoking remains an important public health issue in China, and stronger and more efficient tobacco control is urgently needed.

## Introduction

Smoking is one of the most important avoidable causes of premature deaths and disability globally, causing an estimated six million deaths worldwide in 2010, including one million in China.^1–3^ China now consumes about 40% of the world's total cigarettes, predominantly by men, with a large increase in consumption in urban rather than rural areas over the past three decades.^4^ Since the 1980s, periodic nationally representative surveys of smoking have been undertaken, and showed in 1996 that about two-thirds of men in China ever smoked, and there were over 300 million male smokers, plus 20 million female smokers.^5^ In 2006, China ratified the WHO's Framework Convention on Tobacco Control (FCTC), but its implementation ever since has been slow due to various political, economic and social barriers.^6^

Long-term monitoring of tobacco use in the general population can inform development and evaluation of disease control policies, as advocated by the WHO MPOWER measures,^7^ and help predict the future trend in tobacco-attributed mortality in the general population.^3^ The China Chronic Disease and Risk Factor Surveillance (CCDRFS) was introduced in 2004, aiming to provide a periodic update on the prevalence and secular trend in smoking and other major risk factors (eg, blood pressure, adiposity, blood lipid and diet) that are relevant for non-communicable diseases at the national level.^8–10^ However, the first few surveys were either relatively small^8^ ^9^ ^11^ or had relatively limited information on smoking.^12^

We present the relevant data from the 2010 CCDRFS that covered a large nationally representative sample of ∼100 000 adults aged ≥18 years with detailed information on smoking.^13^ The aim of the present study is to (1) describe the sex-specific prevalence and characteristics of smoking, including cessation in 2010, and to compare that with a similarly large survey in 1996; and (2) estimate the total number of current smokers and number of cigarettes consumed in 2010 in China.

## Methods

### Study population

The details about CCDRFS rational, design, methods and sampling schemes have been described previously.^10^ ^13^ ^14^ In brief, it was based on the China's nationally representative Disease Surveillance Points (DSPs), which was first established in early 1980s and expanded twice in 1989 and 2004, to provide nationally representative mortality and morbidity data in a sample of the general population of mainland China. There are now a total of 161 DSPs and each covers an entire rural county or urban district across all provinces in China, with an estimated total population of 73 million.^15^ The 2010 CCDRFS was conducted in all 161 DSPs from 31 provinces in China (efigure 1).^10^ ^13^

10.1136/jech-2016-207805.supp1 Supplementary data

### Sampling methods

At each DSP, a multistage proportionate to population size sampling scheme was used to select four rural townships or urban neighbourhoods. In each selected township or neighbourhood, 3 administrative villages/communities were further selected randomly and within each of them, 1 residential group with 50 households was selected by simple random sampling. For each selected household, one individual who was ≥18 years of age and had lived locally for at least 6 months was selected by Kish grid.^16^ In a proportion (9%) of the selected household, the individual refused or was unavailable to participate after three attempts by the survey team, and a replacement was made from the households with a similar composition in the same village or community.^13^

### Data collection

In each household, trained health workers measured height, weight, waist circumference and blood pressure; took blood for subsequent measurements of fast and oral glucose tolerance test blood glucose, lipid, insulin and glycated hemoglobin in a central laboratory; and completed a standard questionnaire in response to answers from participants on demographics, smoking, alcohol drinking, physical activity, diet and self-reported medical history.^14^ Survey records were on paper first, with subsequent data entry double-punched. Overall, 108 423 residents were selected and 98 058 (90%) responded, covering all 31 provinces in China.

National and local ethics approvals were obtained prior to start of the project. All participants provided written informed consent.

### Assessment of smoking status

Questions about smoking included frequency, type, amount, age started, intention or attempt to quit (for current smokers), with additional information on knowledge towards smoking cessation and tobacco promotion (see etable 1 for questionnaire). Smokers were those who smoked at least one cigarette or equivalent on most days for at least 6 months. Ex-smokers were those who had stopped ≥6 months at the time of the survey, with those who had quit for ≥2 years defined as successful quitters.^17^ For this study, 178 participants were excluded due to key smoking information missing, leaving 97 880 for the final analyses.

### Statistical analyses

The sex-specific prevalence rate, percentage or mean related to smoking and smoking cessation were calculated both overall and by population subgroups (eg, age or year of birth, geographic areas, education, household income). Appropriate weighting strategies based on a complex sampling design was applied to the prevalence or mean estimates.^10^ ^14^ To address the unequal probability of individual selection, sample selection weight was generated by multiplying the reciprocal of the probability of each individual being selected. To address the deviation of gender and age distribution, the 2010 Chinese census population was used to calculate the poststratification factor, which was multiplied by the sample selection weight to yield the final weighting for each individual. SEs were calculated using the Taylor linearisation method with finite population correction appropriating for the complex sampling design. Rao-Scott χ^2^ tests were conducted to test for differences in prevalence or percentage, and the logistic regression model was used to examine the trends for ordered categorical variables. SAS V.9.4 (SAS Institute, Cary, North Carolina, USA) was used for all analyses performed in 2015.

## Results

### Prevalence and patterns of smoking among men

Among men, 62.4% (60.3% to 64.4%) of adults aged ≥18 years ever smoked, including 54.0% (52.2% to 55.9%) current smokers and 8.4% (7.8% to 9.0%) ex-smokers. The prevalence of current smoking was higher at age 40–59 than other age groups, higher in rural residents and in those with lower education, but varied little by household income (table 1). Large regional variations in smoking prevalence were observed, with over 70% of the male population being ever regular smokers in Yunnan, Guizhou, Qinghai, Hunan, Hainan, Jiangsu, Beijing and Gansu (efigure 1).

**Table 1:** Estimated percentage (%) of tobacco smoking by selected demographic characteristics among Chinese adults in 2010

	Men					Women				
	*N* of participants	Never-smoker (%, 95% CI)	Ever-smoker (%, 95% CI)			*N* of participants	Never-smoker (%, 95% CI)	Ever-smoker (%, 95% CI)		
			All	Ex-smoker	Current smoker			All	Ex-smoker	Current smoker
Overall	44 798	37.6 (35.6 to 39.7)	62.4 (60.3 to 64.4)	8.4 (7.8 to 9.0)	54.0 (52.2 to 55.9)	53 082	96.6 (96.0 to 97.3)	3.4 (2.7 to 4.0)	0.8 (0.6 to 1.0)	2.6 (2.0 to 3.2)
Age group (years)										
18–29	7215	50.6 (47.3 to 54.0)	49.4 (46.0 to 52.7)	2.9 (2.3 to 3.5)	46.5 (43.2 to 49.7)	7446	98.3 (97.9 to 98.8)	1.7 (1.2 to 2.1)	0.5 (0.3 to 0.7)	1.2 (0.8 to 1.6)
30–39	7975	37.5 (34.9 to 40.1)	62.5 (59.9 to 65.1)	4.9 (4.2 to 5.5)	57.6 (55.1 to 60.1)	9865	97.9 (97.3 to 98.5)	2.1 (1.5 to 2.7)	0.3 (0.1 to 0.4)	1.8 (1.3 to 2.5)
40–49	10 874	32.0 (29.8 to 34.3)	68.0 (65.7 to 70.2)	7.7 (6.8 to 8.5)	60.3 (58.2 to 62.3)	13 858	97.4 (96.7 to 98.2)	2.6 (1.8 to 3.3)	0.3 (0.2 to 0.4)	2.3 (1.6 to 2.9)
50–59	9292	29.3 (27.0 to 31.7)	70.7 (68.3 to 73.0)	11.2 (10.2 to 12.1)	59.5 (57.5 to 61.4)	11 562	95.6 (94.5 to 96.8)	4.4 (3.2 to 5.5)	1.0 (0.7 to 1.3)	3.4 (2.4 to 4.3)
60–69	6046	30.3 (27.3 to 33.4)	69.7 (66.6 to 72.7)	17.5 (15.5 to 19.4)	52.2 (49.5 to 54.9)	6649	93.5 (91.7 to 95.4)	6.5 (4.6 to 8.3)	1.7 (1.2 to 2.3)	4.8 (3.3 to 6.2)
70	3396	36.3 (32.6 to 39.9)	63.7 (60.1 to 67.4)	22.2 (20.3 to 24.1)	41.5 (38.1 to 44.9)	3702	91.3 (89.0 to 93.6)	8.7 (6.4 to 11.0)	2.9 (1.8 to 4.0)	5.8 (4.2 to 7.3)
p Value for test for trend		<0.001	<0.001	<0.001	0.4650		<0.001	<0.001	<0.001	<0.001
Area										
Urban	11 779	41.6 (38.4 to 44.9)	58.4 (55.1 to 61.6)	8.8 (7.7 to 9.9)	49.6 (46.8 to 52.3)	15 045	96.4 (95.5 to 97.2)	3.6 (2.8 to 4.5)	1.0 (0.7 to 1.3)	2.6 (1.9 to 3.3)
Rural	33 019	36.1 (33.6 to 38.6)	63.9 (61.4 to 66.4)	8.2 (7.5 to 8.9)	55.7 (53.4 to 58.0)	38 037	96.7 (96.0 to 97.5)	3.3 (2.5 to 4.0)	0.7 (0.5 to 0.9)	2.6 (2.0 to 3.2)
p Value for test for difference		0.0059	0.0059	0.7259	0.0009		0.4822	0.4822	0.1395	0.9108
Education										
None	3317	34.9 (31.6 to 38.3)	65.1 (61.7 to 68.4)	13.7 (12.2 to 15.2)	51.4 (47.8 to 54.8)	10 249	94.3 (92.9 to 95.7)	5.7 (4.3 to 7.1)	1.6 (1.0 to 2.1)	4.1 (3.0 to 5.2)
Primary	12 919	30.0 (27.2 to 32.9)	70.0 (67.1 to 72.8)	11.6 (10.5 to 12.6)	58.4 (55.7 to 61.1)	16 262	95.7 (94.5 to 96.9)	4.3 (3.1 to 5.5)	0.9 (0.6 to 1.1)	3.4 (2.4 to 4.5)
Secondary	24 341	38.2 (35.9 to 40.5)	61.8 (59.5 to 64.1)	7.0 (6.3 to 7.7)	54.8 (52.8 to 56.8)	22 703	97.6 (97.1 to 98.0)	2.4 (2.0 to 2.9)	0.5 (0.4 to 0.7)	1.9 (1.5 to 2.3)
Tertiary	4221	52.5 (49.3 to 55.6)	47.5 (44.4 to 50.7)	6.0 (4.9 to 7.2)	41.5 (38.2 to 44.8)	3868	98.7 (98.2 to 99.3)	1.3 (0.7 to 1.8)	0.4 (0.0 to 0.7)	0.9 (0.5 to 1.3)
p Value for test for trend		<0.001	<0.001	<0.001	<0.001		<0.001	<0.001	<0.001	<0.001
Household income (×1000 CN¥/year)*										
<5	3841	33.1 (28.9 to 37.2)	66.9 (62.8 to 71.1)	12.1 (10.7 to 13.5)	54.8 (50.6 to 59.0)	4539	94.4 (92.4 to 96.2)	5.6 (3.8 to 7.6)	1.6 (0.8 to 2.5)	4.0 (2.8 to 5.3)
5	5523	34.7 (30.5 to 38.9)	65.3 (61.1 to 69.5)	9.4 (8.1 to 10.6)	56.0 (51.7 to 60.2)	6431	96.2 (95.1 to 97.4)	3.8 (2.6 to 4.9)	1.0 (0.6 to 1.4)	2.8 (1.8 to 3.6)
10	9610	36.3 (33.6 to 39.1)	63.7 (60.9 to 66.4)	8.0 (7.1 to 8.9)	55.7 (53.1 to 58.3)	11 756	96.1 (95.2 to 97.0)	3.9 (3.0 to 4.8)	0.7 (0.5 to 0.9)	3.2 (2.4 to 4.0)
20	9968	37.4 (35.0 to 39.9)	62.6 (60.1 to 65.0)	8.2 (7.3 to 9.1)	54.4 (52.1 to 56.6)	11 352	96.5 (95.6 to 97.5)	3.5 (2.5 to 4.4)	0.7 (0.5 to 0.9)	2.8 (1.9 to 3.6)
35	9566	37.9 (35.4 to 40.5)	62.1 (59.5 to 64.6)	8.1 (7.2 to 9.0)	54.0 (51.6 to 56.3)	10 786	97.4 (96.8 to 98.0)	2.6 (2.0 to 3.2)	0.7 (0.4 to 0.9)	1.9 (1.4 to 2.4)
p Value for test for trend		0.0907	0.0907	<0.001	0.5629		0.0015	0.0015	0.0182	0.0025

*6290 male and 8218 female participants refused to provide household income.

**Figure 1:**
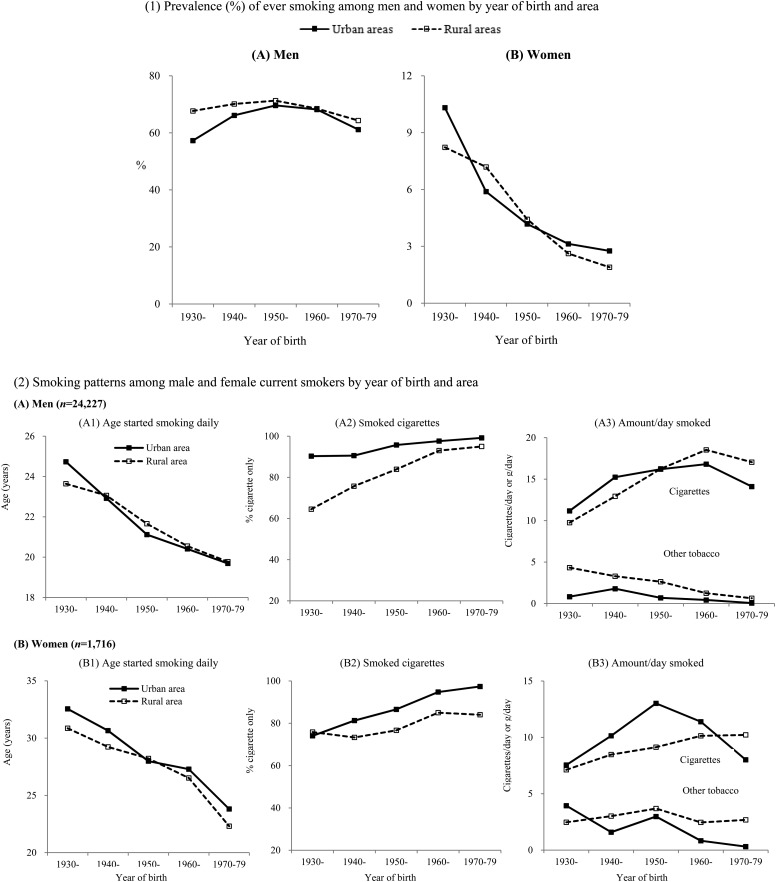
Prevalence (%) of ever smoking and smoking patterns among male and female current smokers by year of birth and area.

In urban and rural men, the smoking prevalence was higher in those born before the 1950s than in those born after the 1950s and the urban–rural difference was seen in each birth cohort, with the difference becoming smaller in those born after the 1950s (figure 1). These patterns were similar both in the low and high prevalence regions (efigure 2).

Among male smokers, the overall mean age to start smoking daily was 20.3 (20.1 to 20.6) years, falling steadily in urban and rural men with later year of birth (23.8 in those born in 1930–1939 vs 21.5 in 1950–1959 vs 19.8 in 1970–1979). Nearly all male current smokers used manufactured cigarettes only (91.7%, 90.0% to 93.4%), higher in urban (97.2%, 96.4% to 98.1%) than in rural areas (89.9%, 87.7% to 92.0%). However, among those born after the 1970s, nearly all smokers in urban and rural areas smoked manufactured cigarettes only (96.1% in 1970–1979 vs 86.8% in 1950–1959 vs 69.0% in 1930–1939). The number of cigarette smoked averaged 15.3 (14.8 to 15.7) cigs/day, higher in rural than urban men (15.6 (15.0 to 16.2) vs 14.2 (13.7 to 14.7)). For any tobacco combined, the mean total consumption per smoker was 16.5 (16.1 to 16.9) g/day (14.7 (14.2 to 15.1) for urban men and 17.1 (16.6 to 17.6) for rural men; figure 1, etable 2).

### Prevalence and patterns of smoking among women

Among women, only 3.4% (2.7% to 4.0%) ever smoked (2.6% (2.0% to 3.2%) current smokers, 0.8% (0.6% to 1.0%) ex-smokers). The smoking prevalence was higher in those with poor education, in urban residents and in those with a lower household income, which is different from men, possibly because income is not positively correlated with education for men and family income mainly comes from men in China. It also increased with increasing age, being 2.1% (1.5% to 2.7%), 2.6% (1.8% to 3.3%), 4.4% (3.2% to 5.5%) and 6.5% (4.6% to 8.3%) at ages 30–39, 40–49, 50–59 and 60–69, reflecting mainly the significant decrease in uptake rates of smoking across successive generations of women (table 1). The prevalence also varied across different provinces, with women in Jilin, Heilongjiang, Liaoning, Inner Mongolia and Tibet having about three times the national average (≥9.0%; efigure 1).

Among female smokers, the mean age first began smoking daily was 26.9 (25.4 to 28.3) years, again falling with the increasing year of birth from 31.3 (28.4 to 34.1) years for those born 1930–1939 to 22.7 (21.6 to 23.9) in those born 1970–1979. Similar patterns and temporal trends for smoking type and amount to those seen in men were also observed (figure 1, etable 2).

### Smoking quitting among men and women

Among current male smokers, the proportion who intended and attempted to quit smoking were 17.3% (15.9% to 18.7%) and 15.6% (14.3% to 16.9%), respectively. The proportions were higher in the younger generation, in those living in urban areas and better educated. The proportion of successful quitters was 10.9% (10.1% to 11.7%), higher in those of old age, lower education, lower household income or living in urban areas (table 2).

**Table 2:** Smoking cessation among male adults for ever-smokers by selected demographic characteristics in China, 2010

	Current smokers								Ever-smokers		
	n	Per cent intent to quit (95% CI)*			Per cent attempt to quit (95% CI)†			n	Per cent quitting successfully (95% CI)‡		
		All (n=24 227)	Urban (n=5874)	Rural (n=18 353)	All (n=24 227)	Urban (n=5874)	Rural (n=18 353)		All (n=29 136)	Urban (n=7312)	Rural (n=21 824)
Overall		17.3 (15.9 to 18.7)	19.3 (17.3 to 21.2)	16.7 (14.9 to 18.4)	15.6 (14.3 to 16.9)	18.5 (16.0 to 21.1)	14.6 (13.1 to 16.1)		10.9 (10.1 to 11.7)	13.0 (11.6 to 14.4)	10.2 (9.3 to 11.1)
p Value for test for urban–rural differences		0.0375			0.0043				0.0002		
Age group (year)											
18–29	3382	21.8 (19.8 to 23.9)	26.4 (22.3 to 30.5)	20.1 (17.8 to 22.4)	21.3 (18.6 to 24.0)	25.6 (20.4 to 30.8)	19.7 (16.6 to 22.8)	3650	3.8 (3.0 to 4.6)	4.4 (2.5 to 6.3)	3.6 (2.8 to 4.5)
30–39	4603	18.5 (16.7 to 20.2)	18.8 (15.0 to 22.7)	18.3 (16.3 to 20.4)	16.0 (14.4 to 17.6)	19.5 (16.0 to 23.0)	14.8 (13.1 to 16.5)	5088	5.9 (5.0 to 6.7)	7.2 (5.6 to 8.8)	5.4 (4.4 to 6.3)
40–49	6428	16.6 (15.0 to 18.3)	16.9 (13.6 to 20.2)	16.6 (14.7 to 18.4)	14.6 (12.8 to 16.3)	14.7 (12.4 to 17.1)	14.5 (12.5 to 16.5)	7416	9.1 (8.1 to 10.1)	9.6 (7.9 to 11.3)	9.0 (7.8 to 10.1)
50–59	5420	15.1 (13.0 to 17.2)	16.1 (13.0 to 19.2)	14.8 (12.4 to 17.2)	13.1 (11.5 to 14.7)	15.2 (13.0 to 17.4)	12.4 (10.4 to 14.4)	6603	13.3 (12.2 to 14.3)	14.4 (12.5 to 16.2)	12.9 (11.6 to 14.1)
60–69	3032	13.2 (11.2 to 15.3)	15.0 (11.0 to 19.1)	12.8 (10.5 to 15.1)	12.5 (10.8 to 14.2)	16.0 (13.2 to 18.9)	11.6 (9.7 to 13.6)	4195	21.2 (18.8 to 23.6)	30.0 (27.1 to 32.8)	18.7 (16.0 to 21.4)
70	1362	10.8 (8.4 to 13.3)	14.2 (9.6 to 18.8)	10.1 (7.2 to 13.0)	8.2 (6.5 to 9.8)	13.3 (9.7 to 16.9)	7.1 (5.3 to 8.9)	2184	30.5 (27.5 to 33.5)	46.4 (38.0 to 54.7)	25.9 (22.8 to 28.9)
p Value for test for trend		<0.0001	<0.0001	<0.0001	<0.0001	<0.0001	<0.0001		<0.0001	<0.0001	<0.0001
Education											
None	1684	11.7 (8.4 to 15.0)	21.6 (12.0 to 31.2)	11.0 (7.6 to 14.4)	10.2 (7.8 to 12.6)	13.7 (6.2 to 21.2)	10.0 (7.3 to 12.6)	2179	16.8 (14.0 to 19.6)	24.6 (15.3 to 33.9)	16.1 (13.3 to 19.0)
Primary	7331	13.0 (11.2 to 14.8)	16.3 (11.7 to 20.9)	12.6 (10.7 to 14.5)	12.5 (11.1 to 13.9)	17.8 (13.8 to 21.7)	11.9 (10.4 to 13.3)	9081	13.6 (12.3 to 14.8)	20.7 (18.0 to 23.4)	12.6 (11.3 to 13.9)
Secondary	13 426	18.6 (17.1 to 20.2)	18.7 (16.4 to 20.9)	18.6 (16.7 to 20.4)	16.4 (14.9 to 17.9)	17.8 (15.5 to 20.1)	15.9 (14.0 to 17.7)	15 731	9.1 (8.2 to 10.0)	11.4 (9.9 to 12.9)	8.2 (7.2 to 9.2)
Tertiary	1786	24.7 (21.6 to 27.8)	22.5 (18.7 to 26.2)	28.5 (23.7 to 33.4)	22.8 (19.2 to 26.3)	21.5 (16.7 to 26.4)	24.9 (20.6 to 29.1)	2145	10.8 (8.4 to 13.1)	12.3 (8.9 to 15.7)	8.1 (5.6 to 10.7)
p Value for test for trend		<0.0001	0.0313	<0.0001	<0.0001	0.0100	<0.0001		<0.0001	<0.0001	<0.0001
Household income (×1000 CN¥/year)§											
<5	2089	15.6 (12.1 to 19.2)	19.4 (10.0 to 28.8)	15.4 (11.7 to 19.1)	12.6 (10.5 to 14.8)	26.7 (16.5 to 36.8)	11.9 (9.6 to 14.1)	2632	13.8 (11.9 to 15.7)	10.1 (3.7 to 16.5)	14.0 (12.1 to 15.9)
5	3089	17.7 (14.3 to 21.0)	21.3 (13.8 to 28.8)	17.4 (13.8 to 20.9)	14.5 (12.2 to 16.9)	17.0 (10.4 to 23.5)	14.3 (11.8 to 16.9)	3721	12.2 (10.3 to 14.1)	12.7 (9.9 to 15.6)	12.2 (10.1 to 14.2)
10	5355	16.8 (14.7 to 18.9)	15.7 (12.7 to 18.7)	17.0 (14.6 to 19.3)	15.8 (13.8 to 17.7)	18.6 (15.5 to 21.7)	15.3 (13.0 to 17.5)	6366	10.2 (9.0 to 11.5)	14.1 (11.7 to 16.5)	9.5 (8.2 to 10.8)
20	5425	17.3 (15.4 to 19.2)	19.3 (15.3 to 23.2)	16.7 (14.5 to 18.8)	15.6 (14.0 to 17.2)	17.1 (14.1 to 20.0)	15.1 (13.2 to 17.0)	6498	10.8 (9.5 to 12.0)	13.9 (11.2 to 16.5)	9.7 (8.4 to 11.0)
35	5035	18.6 (16.8 to 20.4)	19.2 (16.2 to 22.1)	18.1 (15.8 to 20.4)	17.4 (15.3 to 19.4)	17.7 (14.2 to 21.2)	17.1 (15.0 to 19.1)	6105	10.6 (9.4 to 11.7)	13.0 (11.2 to 14.8)	8.4 (7.2 to 9.5)
p Value for test for trend		0.2636	0.5634	0.4394	0.0081	0.3377	0.0043		0.0089	0.9065	<0.0001

*Proportion of intent to quit smoking within 12 months among current smokers.

†Proportion of having attempted to stop smoking among current smokers in the past 12 months.

‡Proportion of having quit smoking over 2 years among ever-smokers.

§In total,3814 ever-smokers refused to provide household income.

Among male ex-smokers, the mean years of quitting smoking were 7.5 (7.0 to 7.9), higher in urban (9.3, 8.5 to 10.0) than in rural men (6.8, 6.3 to 7.3). Among female ex-smokers, the mean quitting duration was 8.2 (7.1 to 9.3) years, with little difference between urban and rural areas (etable 3).

A small proportion of male current (0.9%) and ex-smokers (3.5%) reported having tried nicotine replacement therapy (NRT) or other Western drugs to quit smoking. About half of the current male smokers reported having received general advice about smoking cessation while seeking medical care in hospital (efigure 3).

Over one-quarter of men reported having noticed tobacco promotion advertisement/logo on 1 or more of 10 types of media in the past 30 days, with the proportion higher in younger men, in those living in urban areas or better educated. In contrast, approximately one-third of men, higher in the elderly, in rural areas or those with lower education, reported not having noticed any health warning message about smoking hazards or message related to smoking cessation across all of nine types of media (efigure 4).

### Number of smokers in China in 2010

By applying the smoking prevalence and tobacco consumption from this nationally representative survey to the 2010 China census population, it was estimated that in 2010, 318 million adults (304 million men and 14 million women) were current smokers, consuming a total 1740 billion manufactured cigarettes.

### Comparison with the national survey in 1996

**Figure 2:**
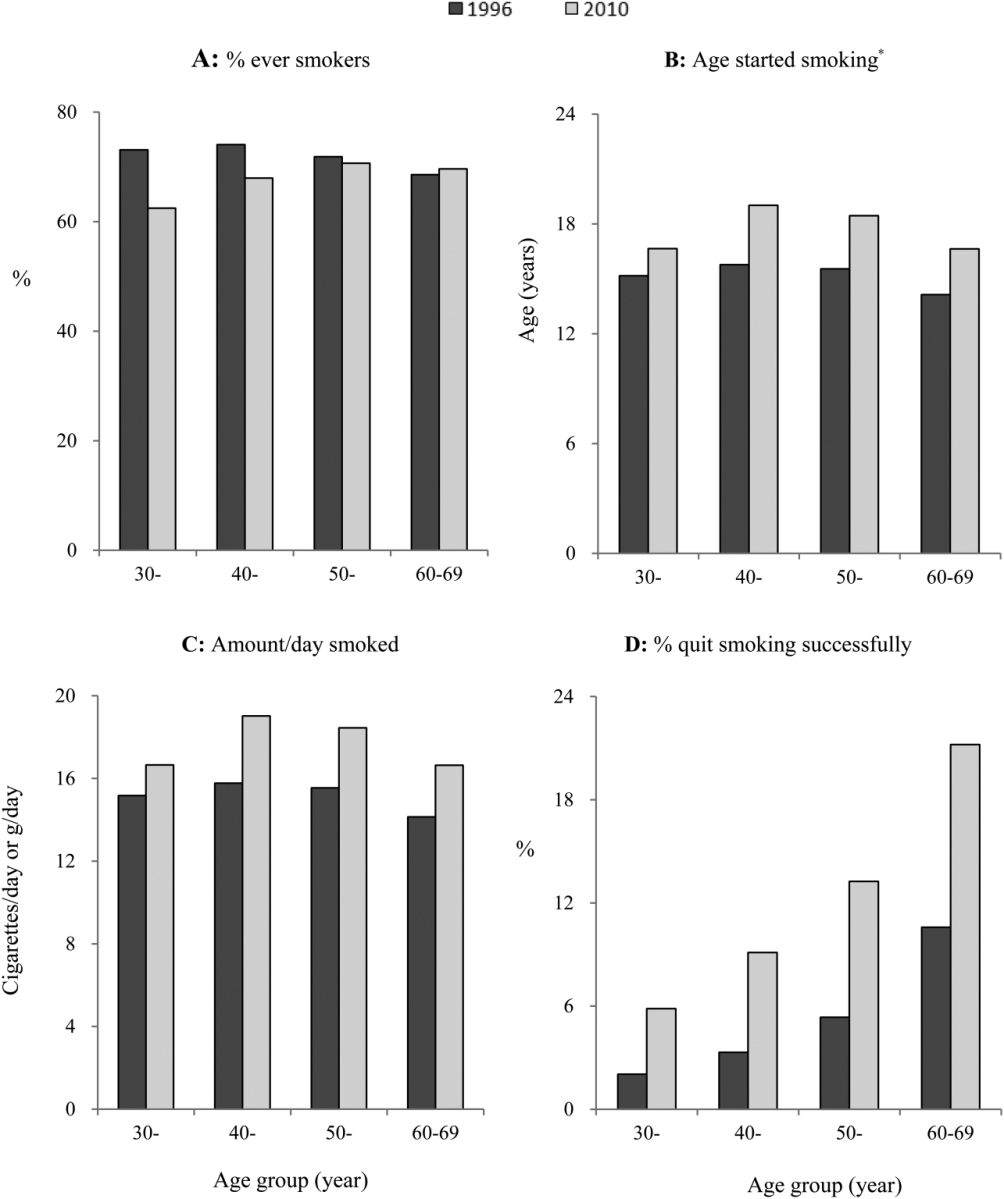
Smoking and quitting smoking among men by age groups, 1996–2010. *Age started was defined as the age to start attempting to smoke the first cigarette among smokers in 1996 survey, while the age to start smoking daily among current smokers in 2010 survey.

Compared with the National Smoking Prevalence Survey (NSPS) in 1996 of 86 770 adults aged 30–69,^5^ the male ever-smoking prevalence was 5.4% lower (67.1% vs 72.5%), mainly at age below 60. There was little difference in mean age to start to smoke (20.6 vs 20.8). However, the amount smoked per smoker increased by 2.7 (15.2 vs 17.9) cigs/day. The proportion of successful quitters had increased from 4.2% in 1996 to 11.0% in 2010, which was evident across all age groups (figure 2).

## Discussion

This large nationally representative survey provided new evidence about the prevalence of smoking in China in the current decade. It showed that about two-thirds of Chinese adult men were ever regular smokers and most of the current smokers, especially those born after 1970, smoked exclusively manufactured cigarettes, consuming on average almost one pack a day. Encouragingly, a higher proportion of male smokers had stopped compared with a decade ago and far fewer Chinese women than men smoked, and there was a large intergenerational decrease in the female prevalence of smoking from about 10% among women born in the 1930s down to only about 2% among women born around the 1970s. In 2010, there were almost 320 million current smokers in China, predominantly men, consuming about 1740 billion cigarettes annually.

Compared with a similar survey in 1996, although the smoking rate had slightly decreased, the number of male current smokers in 2010 had increased mainly due to the population growth. According to China's official cigarette sale figures,^18^ in 2010 China consumed a total of 2300 billion cigarettes, which is 600 billion more than our estimates. The reasons for the discrepancy are not clear. It is possible that our survey using a self-reported questionnaire without biochemical validation (eg, exhaled CO or measures of blood cotinine levels) may underestimate the prevalence of current smokers and/or cigarettes consumption per smokers.^4^ On the other hand, sales data may not necessarily reflect real consumption levels in the population, for in China a proportion of cigarettes are used as gifts without being necessarily smoked.^5^

The recent report from the Global Adult Tobacco Survey (GATS) in 2010 among 14 low-income and middle-income countries showed that China had the highest male smoking prevalence only after Russia. The GATS included 13 354 participants (49% male) aged 15 and above in China and found that 53% of Chinese men were current smokers,^19^ similar to our estimate. Among women, however, the GATS also found that the prevalence of smoking among Chinese women was among the very lowest (2.4%).^19^ Consistent with this and other surveys in China, our study further showed that there was a large intergenerational decrease in the uptake rate of smoking among women over the past several decades. The reasons for the contrasting male and female smoking trends are not clear. However, social changes may well lead young Chinese women to start smoking, as in many Western countries. Indeed, a recent study showed that tobacco use by adolescent females has recently begun to increase in some regions of China.^20^

Our survey showed that Chinese men who were born in the 1970s had a high smoking prevalence and smoked almost exclusively manufactured cigarettes from early adulthood. If the current situation continues, they will probably be the first generation in China to experience the full hazards from tobacco use unless they stop.^21^ Previous large prospective studies in China showed that the hazard associated with a given pattern of smoking was more extreme in urban than in rural areas.^3^ ^21^ ^22^ In China, cigarette consumption became widespread earlier in urban than in rural areas, mainly due to the limited availability of cigarettes in the decades before the 1980s.^3^ However, this urban and rural difference in smoking-attributed hazards is likely to diminish, or even be reversed, over the next few decades, because rural men born after the 1960s tended to start smoking at the same age as urban men and to smoke only cigarettes, which carries a greater risk than traditional forms of tobacco, as well as had a higher smoking prevalence. Successful linkage of periodic NSPS participants with routine mortality records should allow reliable monitoring of the evolution of tobacco-attributed mortality in urban and rural China for many decades to come.

Previous studies in China showed that many smokers in China quit smoking mainly due to poor health.^3^ ^5^ In our survey, no specific information about reasons for quitting was collected. Nevertheless, compared with a similar survey in 1996,^17^ a higher proportion of smokers had quit smoking in 2010. Despite this, however, most current smokers in our survey did not intend to quit, in contrast to those in the USA, the UK and other developed countries,^1^ ^19^ ^23^ as well as many Asian populations.^19^ In a Korean study, two-thirds of smokers intended to quit and 57% of current smokers have ever tried to quit.^24^ The benefits from smoking cessation are highly evident, especially at an earlier age.^17^ ^25^ ^26^ As well as health education to inform the general public about the hazards of smoking, behavioural and social support combined with pharmacotherapy should also be considered as important components of intervention in smoking cessation.^27^ In China, the smoking cessation clinic was introduced in 1996 by the National Health and Family Planning Commission (previously the Ministry of Health) that has now been rapidly expanded in big cities mainly in senior hospitals. Despite this, few smokers sought help from it, partly for NRT excluded in the national insurance list or very low coverage nationwide, and the efforts have not come up to the expectation,^4^ suggesting Chinese government should increase the access to NRT or other western drug therapy usage, and reinforces the media campaigns.

China officially ratified the FCTC in 2006 and ever since progress has been made in tobacco control.^4^ In September 2015, 20 years after the implementation of the old version, a reinforced Advertisements Law was deployed in China to meet the comprehensive bans on tobacco advertising as part of FCTC. The year 2015 also saw the implementation of ‘Tax Price Linkage’ for the first time in China which will increase^28^ the cigarette wholesale and valorem tax rate from 5% to 11%. However, the tax increase still seems very modest compared with that in some developed countries, for example, the price of a pack of cigarettes increased by 44.7% in France during 2003–2004.^29^ The cigarette price in China is still the cheapest worldwide,^28^ although raising tobacco taxation has been confirmed as one of the most effective measures to reduce tobacco consumption that can benefit people's health and households' finances substantially, as well as the country's revenue.^4^ ^30–32^ Implementation of large pictorial health warnings on tobacco packages, as part of the measures recommended by FCTC, is highly cost-effective, but the situation in China is still far from satisfactory and has done little to inform public about the hazards of smoking at present.^4^ More effective and advisable tobacco control advocacy is urgently needed in China, especially for the rural population which is less well educated and does not fully understand the hazards of smoking.

A potential limitation of this study is that all of the smoking-related data were collected by self-report without biochemical validation, which might probably cause information bias, but replication of this design is comparable.^17^ ^33^ Another limitation is the absence of common questions on smoking history for current or/and former smokers to examine whether they have ever smoked for more than 6 months or 100 cigarettes, which has been used to define ever-smokers in the past surveys,^3^ ^5^ and would influence their comparability.

Although the overall smoking prevalence rate is declining, the Chinese male smoking rate is one of the highest worldwide, with a continuously increasing amount of tobacco consumption and lowering age at started smoking. Tobacco smoking remains an important public health issue in China, and stronger and more efficient tobacco control is urgently needed in China, with a priority on those belonging to the younger generation, in rural areas and less educated. Monitoring and evaluation based on large prospective studies and the current nationally representative survey will provide essential and accurate information about the smoking epidemic and tobacco control profile in China.

**What is already known on this subject**

- More than 300 million smokers, predominantly men, make China the largest tobacco consumer in the world, following a huge attributed burden of disease.
- The very high prevalence of smoking in Chinese men, but very low in women, has been reported in a variety of related surveys since 1996, mostly on either a relatively small sample size or limited smoking information.
- The rate of smoking cessation remains very low and most current smokers do not intend to quit in China.

**What this study adds**

- This study, as the third milestone, comprehensively provides the sex-specific patterns of smoking by subgroup based on a large nationally representative sample after 1984 and 1996.
- Despite the smoking prevalence slightly decreasing in Chinese men and women, the amount of cigarette consumption increased and the age to start to smoke became younger compared with that in 1996.
- The men born after 1970, smoking exclusively manufactured cigarettes, will be the first generation to experience the full hazards of cigarette smoking in China.
- There was a large intergenerational decrease with younger age in the female prevalence of smoking.

## References

[1] U.S. Department of Health and Human Services. The health consequences of smoking-50 years of progress: a report of the surgeon general. Atlanta, GA: U.S. Department of Health and Human Services, Centers for Disease Control and Prevention, National Center for Chronic Disease Prevention and Health Promotion, Office on Smoking and Health, 2014.

[2] World Health Organization. Tobacco in China. http://www.wpro.who.int/china/mediacentre/factsheets/tobacco/en/ (accessed 26 May 2015).

[3] Chen Z, Peto R, Zhou M, et al Contrasting male and female trends in tobacco-attributed mortality in China: evidence from successive nationwide prospective cohort studies. Lancet 2015;386:1447–56. 10.1016/S0140-6736(15)00340-2 26466050 PMC4691901

[4] Yang G, Wang Y, Wu Y, et al The road to effective tobacco control in China. Lancet 2015;385:1019–28. 10.1016/S0140-6736(15)60174-X 25784349

[5] Yang G, Fan L, Tan J, et al Smoking in China: findings of the 1996 National Prevalence Survey. JAMA 1999;282:1247–53. 10517427 10.1001/jama.282.13.1247

[6] Hu TW, Lee AH, Mao Z WHO Framework Convention on Tobacco Control in China: barriers, challenges and recommendations. Glob Health Promot 2013;20:13–22. 10.1177/1757975913501910 PMC4041682 24297769

[7] World Health Organization. WHO report on the global tobacco epidemic, 2008: the MPOWER package. Geneva: World Health Organization, 2008.

[8] Chinese Center for Disease Control and Prevention. Report on Chronic Disease Risk Factor Surveillance in China 2004. Beijing: Peking Union Medical College press, 2009.

[9] Chinese Center for Disease Control and Prevention. The report of 2007 Behavioral Risk Factors Surveillance of China. Beijing: People's Medical Publishing House, 2010.

[10] Li Y, Wang L, Jiang Y, et al Risk factors for noncommunicable chronic diseases in women in China: surveillance efforts. Bull World Health Organ 2013;91:650–60. 10.2471/BLT.13.117549 24101781 PMC3790222

[11] Li Q, Hsia J, Yang G Prevalence of smoking in China in 2010. N Engl J Med 2011;364:2469–70. 10.1056/NEJMc1102459 21696322

[12] Qian J, Cai M, Gao J, et al Trends in smoking and quitting in China from 1993 to 2003: National Health Service Survey data. Bull World Health Organ 2010;88:769–76. 10.2471/BLT.09.064709 20931062 PMC2947036

[13] Xu Y, Wang L, He J, et al Prevalence and control of diabetes in Chinese adults. JAMA 2013;310:948–59. 10.1001/jama.2013.168118 24002281

[14] Zhao WH, Ning G, National Workgroup of China Chronic Disease Surveillance. Methodology and content of China chronic disease surveillance (2010). Chin J Prev Med 2012;46:477–9.

[15] Liu S, Wu X, Lopez AD, et al An integrated national mortality surveillance system for death registration and mortality surveillance, China. Bull World Health Organ 2016;94:46–57. 10.2471/BLT.15.153148 26769996 PMC4709796

[16] Kish L A procedure for objective respondent selection within the household. J Am Stat Assoc 1949;44:380–7.

[17] Yang G, Ma J, Chen A, et al Smoking cessation in China: findings from the 1996 national prevalence survey. Tob Control 2001;10:170–4. 11387539 10.1136/tc.10.2.170 PMC1747542

[18] National Bureau of Statistics of China. National data. http://data.stats.gov.cn/ (accessed May 25 2015).

[19] Giovino GA, Mirza SA, Samet JM, et al Tobacco use in 3 billion individuals from 16 countries: an analysis of nationally representative cross-sectional household surveys. Lancet 2012;380:668–79. 10.1016/S0140-6736(12)61085-X 22901888

[20] Han J, Chen X A meta-analysis of cigarette smoking prevalence among adolescents in China: 1981–2010. Int J Environ Res Public Health 2015;12:4617–30. 10.3390/ijerph120504617 25922989 PMC4454929

[21] Liu BQ, Peto R, Chen ZM, et al Emerging tobacco hazards in China: 1. Retrospective proportional mortality study of one million deaths. BMJ 1998;317:1411–22. 9822393 10.1136/bmj.317.7170.1411 PMC28719

[22] Gu D, Kelly TN, Wu X, et al Mortality attributable to smoking in China. N Engl J Med 2009;360:150–9. 10.1056/NEJMsa0802902 19129528

[23] World Health Organization. Tobacco or health: a global status report. Geneva: World Health Organization, 1997.

[24] Kim H, Oh JK, Lim MK, et al The national “smoking cessation clinics” program in the Republic of Korea: socioeconomic status and age matter. Asian Pac J Cancer Prev 2013;14:6919–24. 24377626 10.7314/apjcp.2013.14.11.6919

[25] Jha P, Ramasundarahettige C, Landsman V, et al 21st-century hazards of smoking and benefits of cessation in the United States. N Engl J Med 2013;368:341–50. 10.1056/NEJMsa1211128 23343063

[26] Pirie K, Peto R, Reeves GK, et al Million Women Study Collaborators. The 21st century hazards of smoking and benefits of stopping: a prospective study of one million women in the UK. Lancet 2013;381:133–41. 10.1016/S0140-6736(12)61720-6 23107252 PMC3547248

[27] Kumar R, Prasad R Smoking cessation: an update. Indian J Chest Dis Allied Sci 2014;56:161–9. 25823111

[28] The impacts of tobacco tax increase. http://www.cb.com.cn/special/show/810.html (accessed 25 May 2015).

[29] Lakhdar CB Quantitative and qualitative estimates of cross-border tobacco shopping and tobacco smuggling in France. Tob Control 2008; 17:12–16. 10.1136/tc.2007.020891 18218801

[30] Choi SE Are lower income smokers more price sensitive? The evidence from Korean cigarette tax increases. Tob Control 2016;25:141–6. 10.1136/tobaccocontrol-2014-051680 25430738

[31] Lee JM, Hwang TC, Ye CY, et al The effect of cigarette price increase on the cigarette consumption in Taiwan: evidence from the National Health Interview Surveys on cigarette consumption. BMC Public Health 2004;4:61 10.1186/1471-2458-4-61 15598345 PMC539350

[32] Verguet S, Gauvreau CL, Mishra S, et al The consequences of tobacco tax on household health and finances in rich and poor smokers in China: an extended cost-effectiveness analysis. Lancet Glob Health 2015;3:e206–16. 10.1016/S2214-109X(15)70095-1 25772692

[33] Zhang Q, Li L, Smith M, et al Exhaled carbon monoxide and its associations with smoking, indoor household air pollution and chronic respiratory diseases among 512,000 Chinese adults. Int J Epidemiol 2013;42:1464–75. 10.1093/ije/dyt158 24057999 PMC3807615

