# Supplementary material for: Prevalence and patterns of tobacco smoking among Chinese adult men and women: findings of the 2010 national smoking survey
Source: J Epidemiol Community Health. 2016 Sep 22;71(2):154–61. doi: 10.1136/jech-2016-207805 (PMC5284482; doi:10.1136/jech-2016-207805)
Supplement: Supplementary data [file jech-2016-207805supp_appendices.pdf]

Supplementary online appendices

eFigure 1. Geographical distribution of Disease Surveillance Points (DSP) system and variations in smoking rate among 31 provinces of mainland China by gender, 2010

eTable 1. Questionnaire related to smoking in the China Chronic Disease and Risk Factor Surveillance survey 2010

eFigure 2. Prevalence of ever smoking among men and women by year of birth and area in two categories of provinces, 2010

eTable 2. Mean and percentage of age started daily smoking for current daily smokers and of tobacco consumption (cigarettes plus other tobacco) for current smokers by area and age group among male and female adults, 2010

eTable 3. Mean and percentage of quitting period (years) for male and female ex-smokers by area and age group, 2010

eFigure 3. Approach attempted and external impetus to quit smoking for ever smokers among male adults by age group, education and area.

eFigure 4. Proportion of tobacco promotion and having noticed the message on tobacco control advocacy among male adults by age group, education and area.

eTable 1: Questionnaire related to smoking in the China Chronic Disease and Risk Factor Surveillance survey 2010

No.	Questions	Answers
S1	Do you currently smoke tobacco on a daily basis, less than daily or not at all?	1. Yes, smoke daily<br>2. Yes, smoke non-daily (skip to S3)<br>3. Not at all (skip to S12)
S2	How old were you when you first started smoking daily?	_____years (age)
S3	How much do you normally smoke following types of tobacco?	a. Manufactured: a1___(per day) or a2___(per week)<br>b. Hand-rolled: b1___(per day) or b2___(per week)<br>c. Pipe: c1___(per day) or c2___(per week)<br>d. Cigar: d1___(per day) or d2___(per week)<br>e. Other: e1___(per day) or e2___(per week)
S4	Did you quit smoking in the past? (quit smoking means consider seriously and had some actions to quit)	1. Yes<br>2. No (skip to S7)
S5	During the past 12 months, have you tried to stop smoking?	1. Yes<br>2. No (skip to S7)
S6	In the past 12 months, have you tried to quit smoking by nicotine replacement therapy or other western drugs?	1. Yes<br>2. No
S7	Which of the following was your thought on quitting smoking?	1. Intend to quit within 1 month<br>2. Intend to quit within 12 months<br>3. Intend to quit 12 months later<br>4. Don't intend to quit<br>99. No idea
S8	Have you visited a doctor or other health care provider in the past 12 months?	1. Yes<br>2. No (skip to S10)
S9	During any visit to a doctor or other health care provider, were you advised to quit smoking tobacco?	1. Yes<br>2. No
S10	In the last 30 days, did you notice any health warnings on cigarette packages?	1. Yes<br>2. No (skip to S12)
S11	In the last 30 days, have warning labels on cigarette packages led you to think about quitting?	1. Yes<br>2. No<br>99. No idea<br>(please skip to S17)
S12	In the past, did you smoke?	1. Yes, smoke daily<br>2. Yes, smoke non-daily<br>3. Not at all (skip to S17)<br>99. No idea (skip to S17)
S13	How long has it been since you stop smoking?	___years or ___months or ___weeks or ___days<br>(if more than 1 year or 12 months, please skip to S17)

S14	Have you visited a doctor or other health care provider in the past 12 months?	1. Yes<br>2. No (skip to S16)
S15	During any visit to a doctor or other health care provider, were you advised to quit smoking tobacco?	1. Yes<br>2. No
S16	In the past 12 months, have you tried to quit smoking by nicotine replacement therapy or other western drugs?	1. Yes<br>2. No
S17	In the past 30 days, have you noticed information about the dangers of smoking cigarettes or that encourages quitting in the following medias?	1. Newspaper/magazine; 2. TV; 3. Broadcast; 4. Bulletin board; 5. Poster/leaflet; 6.Wall ad; 7. Cinema; 8. Internet; 9. Public transportation or its station
S18	In the past 30 days, have you noticed any advertisements or signs promoting cigarettes in the following medias?	1. Shop selling cigarettes; 2. Newspaper/magazine; 3. TV; 4. Broadcast; 5. Bulletin board; 6. Poster/leaflet; 7. Wall ad; 8. Cinema; 9. Internet; 10. Public transportation or its station

eFigure 1 Geographical distribution of Disease Surveillance Points (DSP) system and variations in smoking rate among 31 provinces of mainland China by gender, 2010

(A) Men **(B) Women**

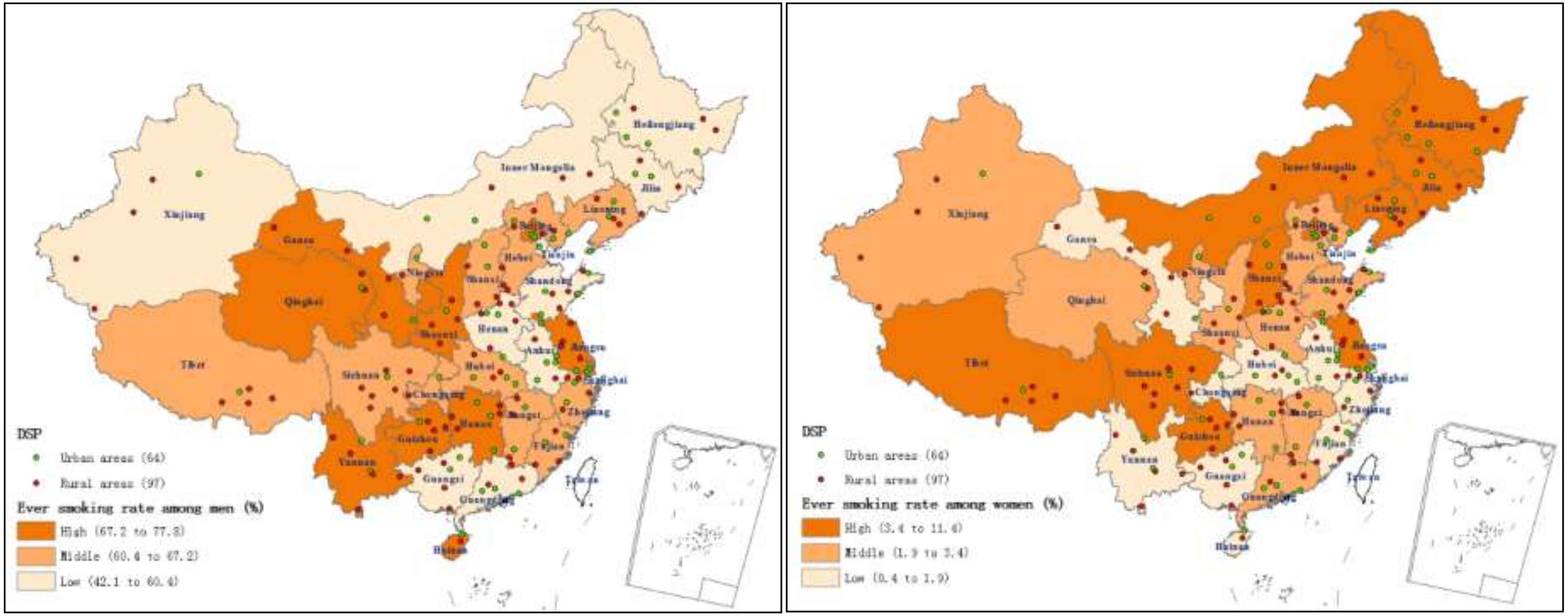

The smoking rates were divided into tertiles representing “high”, “middle” and “low” among 31 provinces separately in men and women.

The provinces with high smoking rate among men include Yunnan (77.7%), Guizhou (77.1%), Qinghai (74.8%), Hunan (74.2%), Hainan (71.5%), Jiangsu (71.5%), Beijing (70.6%), Gansu (70.5%), Shaanxi (69.9%), and Shanghai (67.3%); The provinces with high smoking rate among women include Jilin (11.4%), Heilongjiang (10.8%), Liaoning (10.1%), Inner Mongolia (9.5%), Tibet (9.0%), Shanxi (5.0%), Guizhou (4.6%), Sichuan (4.3%), Jiangsu (4.2%), and Beijing (3.8%).

eFigure 2 Prevalence of ever smoking among men and women by year of birth and area in two categories of provinces, 2010

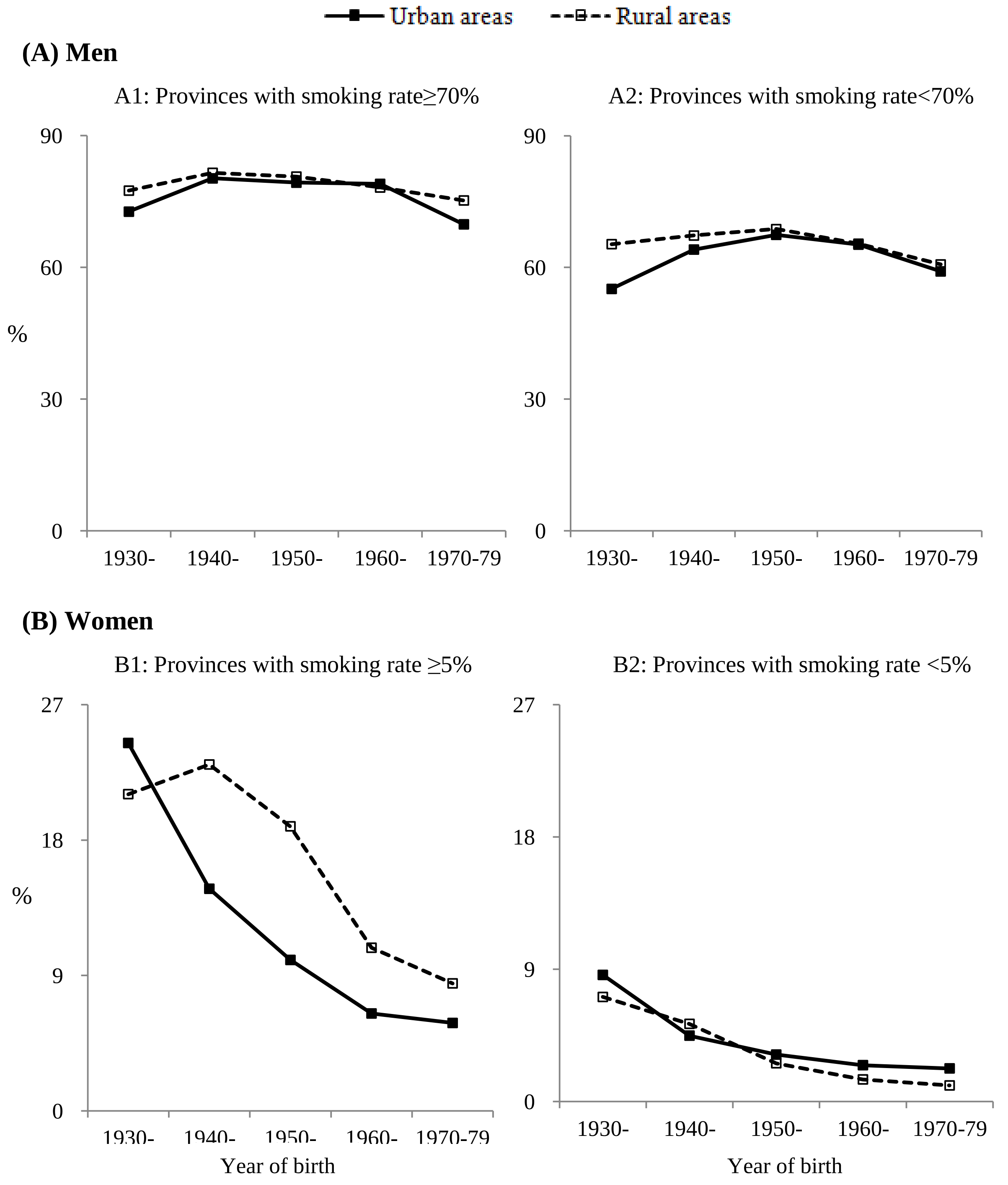

(A) Provinces with smoking rate ≥ 70% among men include Yunnan (77.7%), Guizhou (77.1%), Qinghai (74.8%), Hunan (74.2%), Hainan (71.5%), Jiangsu (71.5%), Beijing (70.6%), Gansu (70.5%), and the rests account for the provinces with smoking rate < 70%.

(B) Provinces with smoking rate ≥ 5% among women include Jilin (11.4%), Heilongjiang (10.8%), Liaoning (10.1%), Inner Mongolia (9.5%), Tibet (9.0%) and Shanxi (5.0%), and the rests account for the the provinces with smoking rate < 5%.

eTable 2 Mean and percentage of age started daily smoking for current daily smokers and of tobacco consumption (cigarettes plus other tobacco) for current smokers by area and age group among male and female adults, 2010

		Age group (years)	Age started daily smoking (95% CI)					Tobacco consumption (95% CI)[a]				
			n	Mean (years)	% of <age 20	% of <age 25	% of ≥age 25	*n*	Mean (years)	% of < 10 cigs/d	% of < 20 cigs/d	% of ≥ 20 cigs/d
Men	Urban	Total	4761	20.2 (19.8-20.5)	46.0 (43.0-49.0)	40.3 (38.3-42.2)	13.7 (11.8-15.6)	5846	14.7 (14.2-15.1)	26.4 (23.9-28.8)	30.5 (27.9-33.2)	43.1 (40.5-45.7)
		18-29	610	18.1 (17.8-18.4)	67.2 (59.9-74.5)	30.8 (23.4-38.1)	2.1 (1.2-3.0)	833	10.7 (9.8-11.5)	41.9 (36.2-47.5)	36.6 (31.6-41.6)	21.5 (17.3-25.8)
		30-39	843	19.7 (19.4-20.1)	46.8 (43.4-50.3)	43.3 (40.6-46.0)	9.9 (6.6-13.2)	1066	14.1 (13.3-14.9)	25.8 (22.8-28.7)	32.8 (29.2-36.4)	41.5 (36.6-46.3)
		40-49	1331	20.4 (19.8-21.0)	41.3 (36.8-45.8)	43.4 (40.0-46.7)	15.4 (12.0-18.7)	1604	16.9 (16.3-17.5)	18.7 (16.2-21.2)	26.1 (22.3-29.8)	55.3 (51.1-59.4)
		50-59	1194	21.0 (20.5-21.5)	38.2 (34.3-42.2)	41.5 (37.0-45.9)	20.3 (16.8-23.9)	1399	16.9 (16.1-17.6)	18.0 (15.9-20.0)	28.0 (24.9-31.1)	54.1 (50.4-57.7)
		60-69	566	22.8 (22.2-23.5)	26.6 (22.8-30.3)	45.4 (40.5-50.2)	28.0 (23.8-32.3)	671	17.0 (15.6-18.5)	19.8 (17.0-22.6)	28.0 (23.4-32.5)	52.3 (47.7-56.9)
		70-	217	24.3 (22.5-26.1)	25.5 (17.9-33.2)	41.2 (33.7-48.7)	33.3 (25.4-41.2)	273	13.0 (12.0-14.0)	35.3 (27.5-43.1)	25.0 (19.9-30.1)	39.7 (34.4-44.9)
	Rural	Total	15617	20.4 (20.1-20.7)	44.9 (42.3-47.5)	39.0 (37.2-40.8)	16.1 (14.5-17.8)	18271	17.1 (16.6-17.6)	20.3 (18.6-22.0)	25.6 (24.0-27.1)	54.2 (51.9-56.4)
		18-29	2079	17.8 (17.6-18.1)	71.0 (67.7-74.3)	27.2 (24.3-30.1)	1.8 (0.9-2.7)	2530	13.1 (12.3-13.9)	31.2 (27.3-35.2)	31.3 (28.7-33.9)	37.5 (33.1-41.9)
		30-39	3010	19.7 (19.4-20.0)	46.2 (43.3-49.1)	41.7 (39.4-44.0)	12.1 (9.9-14.3)	3511	17.5 (16.9-18.2)	16.8 (14.5-19.1)	24.5 (22.0-27.0)	58.7 (55.5-61.8)
		40-49	4138	20.5 (20.3-20.7)	39.7 (36.7-42.7)	44.0 (41.6-46.3)	16.4 (14.5-18.2)	4807	19.7 (19.1-20.4)	13.9 (12.2-15.5)	20.9 (19.2-22.7)	65.2 (62.8-67.7)
		50-59	3496	21.6 (21.2-21.9)	33.1 (30.2-36.0)	42.8 (40.0-45.6)	24.1 (21.4-26.9)	3997	19.0 (18.4-19.6)	15.2 (13.4-17.1)	23.2 (20.8-25.6)	61.5 (58.7-64.4)
		60-69	1992	22.9 (22.4-23.5)	29.0 (25.7-32.4)	39.7 (36.5-43.0)	31.2(27.8-34.7)	2344	16.6 (15.8-17.3)	22.2 (19.4-25.1)	27.2 (24.6-29.7)	50.6 (47.3-54.0)
		70-	902	23.9 (22.8-25.0)	31.7 (27.7-35.7)	35.8 (31.8-39.7)	32.5 (27.2-37.9)	1082	14.2 (13.3-15.1)	32.6 (28.1-37.1)	32.2 (28.7-35.7)	35.2 (30.6-39.8)
Women	Urban	Total	316	27.1 (25.3-28.9)	19.9 (12.9-26.9)	27.7 (20.8-34.5)	52.5 (41.6-63.3)	457	11.2 (9.7-12.7)	44.8 (35.9-53.6)	27.9 (19.9-36.0)	27.3 (21.3-33.2)
		18-29	14	19.5 (18.8-20.1)	48.3 (17.5-79.1)	51.7 (20.9-82.5)	0.0 (0.0-0.0)	27	8.6 (5.0-12.2)	55.8 (31.3-80.3)	23.6 (3.3-43.9)	20.6 (1.5-39.7)
		30-39	41	23.5 (21.0-26.0)	17.2 (2.9-31.4)	42.3 (23.3-61.3)	40.5 (11.7-69.3)	68	7.9 (5.8-10.0)	56.8 (42.5-71.0)	30.3 (16.0-44.6)	12.9 (6.2-19.7)
		40-49	66	27.1 (25.3-29.0)	10.5 (3.4-17.6)	27.7 (13.0-42.3)	61.9 (48.0-75.8)	103	12.2 (10.0-14.4)	38.9 (27.4-50.4)	25.6 (16.0-35.2)	35.5 (26.0-45.0)
		50-59	88	28.0 (24.5-31.4)	22.4 (8.0-36.9)	20.4 (9.9-30.8)	57.2 (40.8-73.7)	114	15.7 (11.9-19.4)	26.8 (17.7-35.9)	29.1 (21.0-37.3)	44.1 (33.3-54.9)
		60-69	61	29.3 (26.9-31.7)	17.0 (5.6-28.4)	18.5 (7.1-29.8)	64.5 (54.2-74.8)	76	12.4 (11.2-13.6)	33.2 (22.8-43.6)	44.6 (27.6-61.6)	22.2 (13.2-31.2)
		70-	46	34.2 (31.9-36.5)	6.9 (1.6-12.1)	10.4 (3.1-17.7)	82.7 (73.7-91.8)	69	10.2 (7.7-12.7)	56.7 (40.3-73.0)	17.9 (9.6-26.1)	25.5 (10.8-40.1)
	Rural	Total	1002	26.8 (25.1-28.5)	27.3 (20.6-34.0)	24.5 (20.0-29.1)	48.1 (40.1-56.2)	1251	11.7 (10.2-13.2)	41.8 (36.1-47.5)	30.5 (26.0-35.0)	27.7 (23.1-32.3)
		18-29	43	18.3 (17.2-19.5)	64.4 (43.9-85.0)	31.2 (10.9-51.4)	4.4 (0.0-9.7)	65	9.7 (6.5-12.8)	51.8 (30.5-73.2)	18.8 (4.7-32.8)	29.4 (13.6-45.2)

30-39	124	21.7 (20.6-22.9)	40.1 (31.6-48.6)	30.7 (21.9-39.4)	29.2 (18.7-39.7)	160	13.3 (10.7-15.9)	32.1 (20.3-43.9)	34.3 (23.7-45.0)	33.6 (24.1-43.1)
40-49	224	26.5 (24.7-28.2)	19.8 (11.7-27.9)	26.7 (20.3-33.0)	53.5 (44.9-62.2)	276	12.6 (10.2-14.9)	35.5 (27.3-43.8)	31.5 (23.8-39.1)	33.0 (23.0-43.0)
50-59	270	28.5 (26.5-30.5)	18.8 (12.0-25.6)	24.1 (18.9-29.3)	57.1 (47.8-66.4)	329	12.7 (10.7-14.7)	36.1 (29.0-43.2)	33.5 (27.6-39.4)	30.4 (23.7-37.2)
60-69	213	28.4 (25.6-31.1)	21.5 (10.3-32.7)	22.6 (15.8-29.3)	56.0 (43.9-68.0)	259	11.6 (9.9-13.2)	42.2 (32.6-51.8)	31.1 (23.5-38.7)	26.7 (18.8-34.5)
70-	128	31.4 (27.8-35.0)	24.2 (12.9-35.4)	17.1 (10.3-23.9)	58.8 (44.7-72.8)	162	9.6 (8.3-11.0)	55.7 (47.0-64.4)	29.3 (21.6-37.0)	15.0 (8.2-21.8)

[a] All types of tobacco were converted to cigarettes.
CI, confidence interval.
cigs/d, cigarettes/day per smoker.

eTable 3 Mean and percentage of quitting period (years) for male and female ex-smokers by area and age group, 2010

Sex	Area	Age group (year)	*n*	Mean (95% CI)	% of quitting period (95% CI)			
					< 0.5	< 1	< 2	≥2
Men	Urban	Total	1438	9.3 (8.5-10.0)	7.8 (6.2-9.3)	4.9 (3.7-6.0)	7.9 (6.0-9.9)	79.5 (76.9-82.0)
		18-29	77	2.8 (1.9-3.6)	17.2 (8.1-26.4)	8.4 (2.7-14.0)	15.3 (5.2-25.3)	59.1 (43.5-74.8)
		30-39	116	5.0 (3.8-6.1)	8.0 (2.7-13.3)	4.5 (1.0-8.0)	13.2 (3.3-23.1)	74.3 (64.7-83.9)
		40-49	255	7.2 (6.3-8.1)	11.7 (7.2-16.3)	6.1 (3.1-9.0)	7.7 (3.5-11.8)	74.5 (68.9-80.2)
		50-59	315	9.4 (8.4-10.4)	6.2 (3.7-8.8)	6.4 (3.1-9.6)	5.5 (3.4-7.7)	81.9 (77.9-85.9)
		60-69	378	11.5 (10.3-12.7)	4.4 (0.7-8.1)	3.7 (1.4-6.0)	6.5 (3.8-9.2)	85.4 (80.5-90.3)
		70-	297	14.7 (13.1-16.2)	4.0 (1.2-6.9)	1.9 (0.0-3.9)	5.0 (1.0-8.9)	89.1 (84.6-93.5)
	Rural	Total	3471	6.8 (6.3-7.3)	12.1 (10.5-13.6)	6.5 (5.3-7.6)	11.7 (10.1-13.2)	69.8 (67.5-72.1)
		18-29	191	2.4 (1.6-3.2)	20.6 (13.9-27.3)	10.7 (4.5-16.9)	19.6 (12.3-26.8)	49.2 (41.3-57.0)
		30-39	369	4.3 (3.7-4.9)	21.9 (16.9-26.9)	7.3 (3.1-11.4)	14.1 (8.8-19.3)	56.8 (50.4-63.1)
		40-49	733	6.0 (5.4-6.6)	10.4 (7.3-13.6)	7.3 (5.1-9.4)	10.9 (7.9-13.9)	71.4 (67.4-75.4)
		50-59	868	7.6 (6.9-8.3)	9.9 (7.4-12.3)	5.7 (3.8-7.5)	10.2 (7.8-12.6)	74.3 (71.1-77.6)
		60-69	785	8.1 (7.1-9.2)	8.5 (6.2-10.9)	6.1 (4.0-8.1)	9.7 (6.7-12.7)	75.7 (71.5-79.9)
		70-	525	9.9 (8.8-10.9)	7.8 (5.6-9.9)	3.9 (1.7-6.1)	10.0 (7.1-13.0)	78.3 (74.3-82.3)
Women	Urban	Total	157	8.3 (6.6-10.0)	6.8 (1.5-12.1)	4.0 (1.1-6.8)	6.1 (1.5-10.8)	83.2 (75.9-90.4)
		18-29	13	3.1 (2.1-4.2)	0.0 (0.0-0.0)	3.2 (0.0-9.8)	11.9 (0.0-29.6)	84.9 (65.8-100.0)
		30-39	14	7.3 (3.9-10.7)	18.3 (0.0-40.9)	6.8 (0.0-20.3)	0.0 (0.0-0.0)	74.9 (49.8-100.0)
		40-49	17	11.6 (4.2-19.0)	6.7 (0.0-16.9)	0.0 (0.0-0.0)	1.4 (0.0-4.3)	91.9 (81.1-100.0)
		50-59	40	7.7 (5.2-10.1)	6.8 (0.0-15.4)	12.7 (1.3-24.0)	7.4 (0.0-16.9)	73.2(52.0-94.4)
		60-69	29	9.5 (5.5-13.4)	19.6 (0.0-43.1)	0.0 (0.0-0.0)	3.1 (0.0-9.5)	77.4 (53.4-100.0)
		70-	44	11.7 (8.2-15.2)	2.0 (0.0-6.1)	1.2 (0.0-3.6)	5.7 (1.0-10.4)	91.1 (84.3-98.0)
	Rural	Total	318	8.2 (6.9-9.5)	13.0 (7.8-18.2)	5.1 (0.6-9.6)	10.0 (6.4-13.6)	71.9 (65.1-78.7)
		18-29	20	4.1 (2.6-5.6)	12.6 (0.0-30.1)	22.3 (0.0-49.2)	0.7 (0.0-1.7)	64.4 (37.3-91.4)
		30-39	18	5.7 (3.2-8.2)	9.0 (0.0-21.7)	3.2 (0.0-9.4)	15.2 (0.0-42.8)	72.6 (41.5-100.0)
		40-49	33	4.9 (2.8-6.9)	24.9 (3.9-45.9)	8.5 (0.0-17.6)	3.7 (0.0-9.1)	62.9 (41.5-84.3)
		50-59	86	7.1 (5.2-9.1)	13.6 (4.7-22.4)	1.9 (0.0-5.1)	12.4 (4.4-20.4)	72.1 (62.2-81.9)
		60-69	93	6.0 (4.1-7.8)	16.0 (10.3-21.7)	1.5 (0.0-3.8)	18.5 (5.7-31.3)	64.0 (49.8-78.2)
		70-	68	14.1 (10.7-17.5)	7.1 (1.8-12.2)	3.8 (0.0-9.7)	4.6 (0.0-10.9)	84.6 (75.4-93.7)

CI, confidence interval.

eFigure 3 Approach attempted and external impetus to quit smoking for ever smokers among male adults by age group, education and area

■ Urban □ Rural

(A) Proportion of having tried nicotine replacement therapy (NRT) or western drug therapy to quit smoking among male ever smokers by age group, education and area

(**A1**) Current smokers (**A2**) Ex-smokers

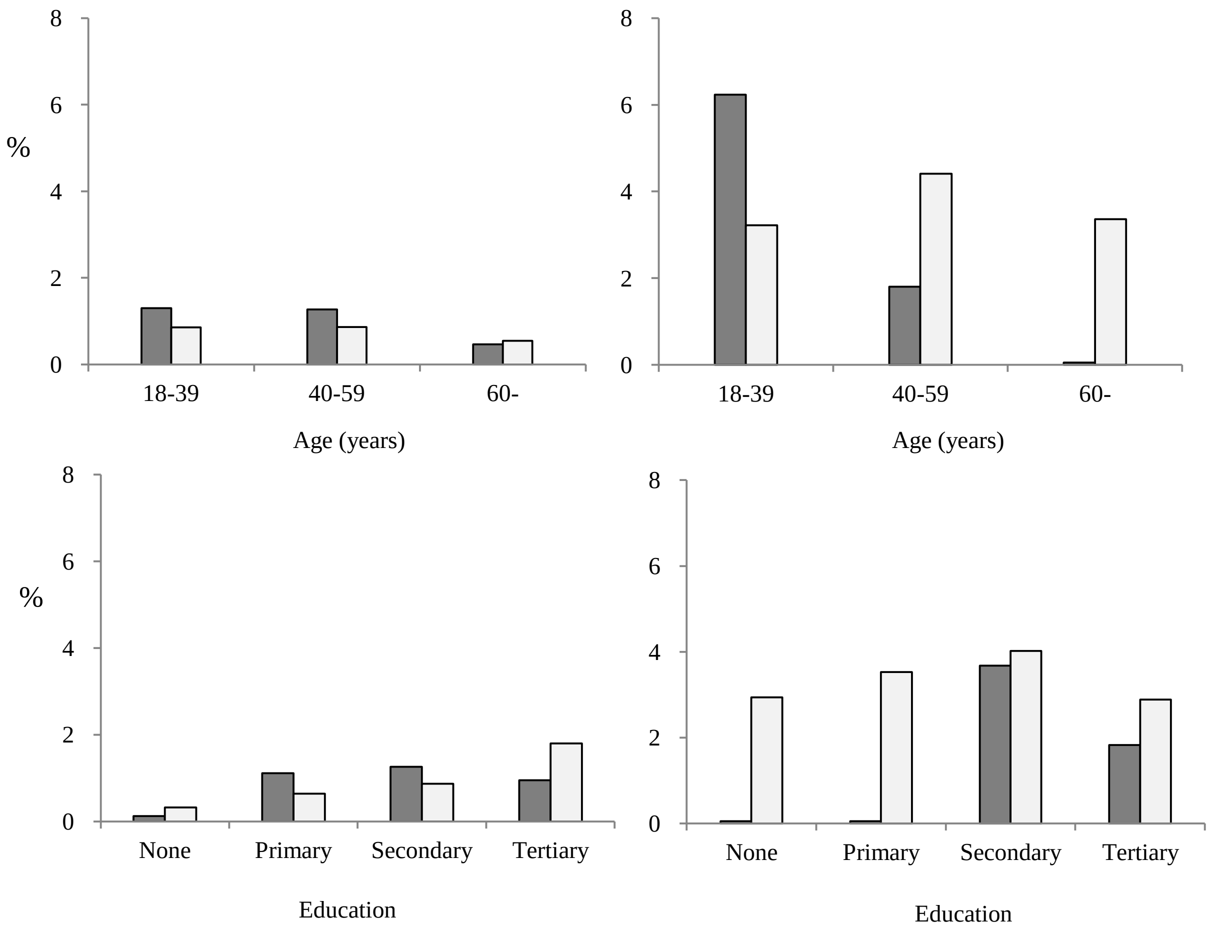

(B) Proportion of being advised to quit smoking for current male smokers who had visited health care providers in the past 12 months

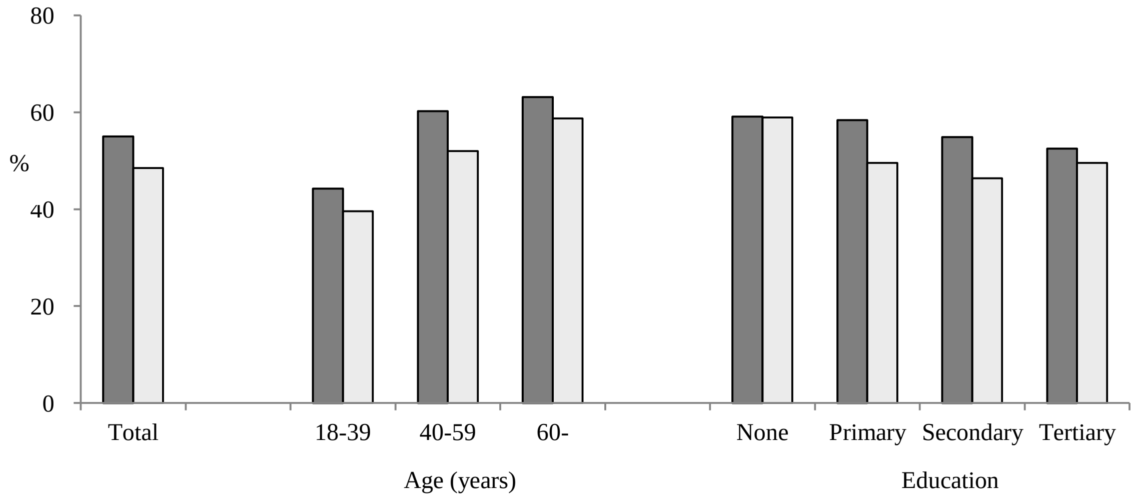

eFigure 4 Proportion of having noticed the tobacco promotion and having not noticed the message on tobacco control advocacy among male adults by age group, education and area

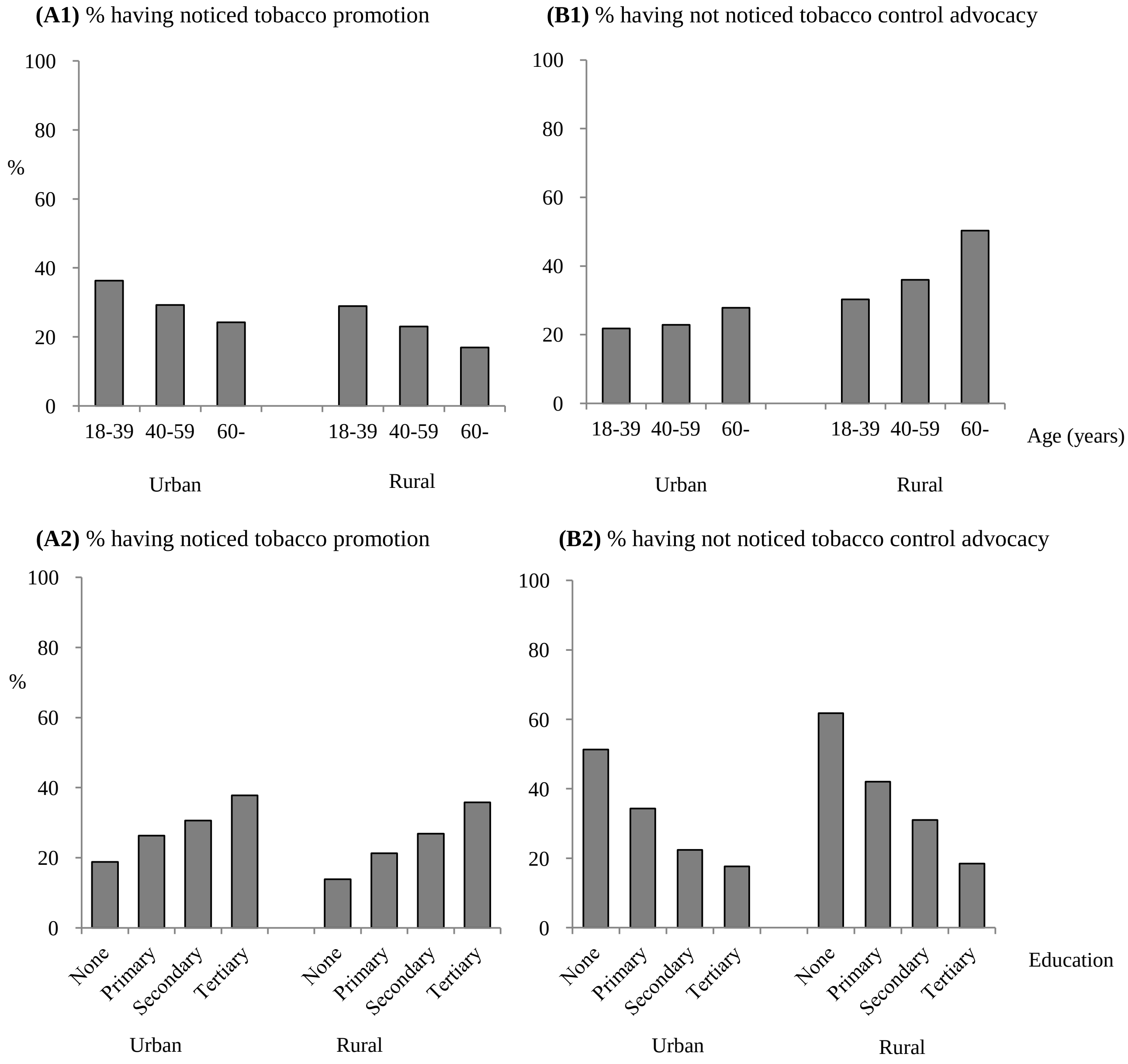

(A1-A2) Proportion of having noticed tobacco promotion ad/logo on one or more types of media or public places in the past 30 days: (1) Shop selling cigarettes; (2) Newspaper/magazine; (3) TV; (4) Broadcast; (5) Bulletin board; (6) Poster/leaflet; (7) Wall ad; (8) Cinema; (9)Internet; (10) Public transportation or its station.
(B1-B2) Proportion of having not noticed the message warning about the dangers of tobacco use or encourage smokers to quit on all 9 types of media or public places in the past 30 days: (1) Newspaper/magazine; (2) TV; (3) Broadcast; (4) Bulletin board; (5) Poster/leaflet; (6) Wall ad; (7) Cinema; (8) Internet; (9) Public transportation or its station.
